# Feeding and developmental outcomes of infants in a South African community

**DOI:** 10.1017/S1463423620000067

**Published:** 2020-03-25

**Authors:** Bronwyn Eales, Esedra Krüger, Marien Graham, Jeannie van der Linde

**Affiliations:** 1 Department of Speech-Language Pathology and Audiology, University of Pretoria, Pretoria, South Africa; 2 Department of Science, Mathematics, and Technology Education, University of Pretoria, Pretoria, South Africa

**Keywords:** early intervention, infant development, infant feeding, lower-middle-income countries, resource-limited community, South Africa

## Abstract

**Background:**

Prevalent environmental risk factors place infants in lower-middle-income countries (LMICs) at an increased risk for feeding and developmental difficulties.

**Aim:**

This study aimed to determine the relationship between feeding and developmental outcomes in infants, as early feeding difficulties may have a cascading effect on developmental outcomes and vice versa.

**Methods:**

Data on 144 infants’ feeding and development [mean age (standard deviation) = 8.8 months (2.2)] from a primary health care clinic in Gauteng, South Africa were retrospectively analysed.

**Results:**

Early introduction of cup feeding was found to be a predictor of possible expressive language and articulation difficulties. Gagging, spitting, or vomiting, pocketing, the use of force feeding, and poor sucking and chewing abilities were significantly associated with behavioural and social–emotional difficulties. Breastfeeding was found to be a protective factor for language development. The results emphasise the importance of primary prevention and early identification of risks in late infancy in LMIC.

Infants from lower-middle-income countries (LMICs) are more adversely affected by biological and psychosocial risks than infants from high-income countries (Lu *et al.*, 2016). South Africa, an LMIC, is characterised by poverty, which is associated with family stress, child abuse or neglect, food insecurity, and exposure to violence (Black *et al.*, 2016). An estimated 25% of South Africans are living in extreme poverty and 56% are living under the poverty line (Statistics South Africa, 2017). Extreme poverty may lead to inappropriate feeding environments, as well as the presence of hostility and disorganisation, often creating a delay or impairment in feeding and developmental outcomes of an infant (Aldridge *et al.*, 2010; Daelmans *et al.*, 2016).

Approximately 56% of children in LMIC are at risk of poor developmental outcomes (Lu *et al.*, 2016) due to the combined effect of poverty and other risk factors. Environmental risks, such as economic disadvantage, housing status, age of the mother, and number of siblings may influence patterns of interaction between the family and infant, creating a disruption in parent–child transactions, family-orchestrated experiences, and health and safety provided by the family (Guralnick, 2013). These environmental risks are associated with delayed language, social, and cognitive development in infants, highlighting the importance of early intervention (EI) for general developmental outcomes in LMICs (Samuels *et al.*, 2012; Guralnick, 2013; Van der Linde *et al.*, 2016). South Africa also has a high prevalence of biological risks, such as preterm birth, low birth weight, Human Immunodeficiency Virus/Acquired Immune Deficiency Syndrome (HIV/AIDS), Foetal Alcohol Spectrum Disorder, and Neonatal Abstinence Syndrome, that lead to feeding and developmental delays in infants (Blencowe *et al.*, 2012; WHO, 2012; Olivier *et al.*, 2016; UNAIDS, 2016; Weich *et al.*, 2017).

Successful growth in early childhood is subject to the complex relationship between sociocultural, biomedical, and physiological processes present during feeding (Berlin *et al.*, 2011). The cascading effect of early feeding difficulties affecting growth and nutrition is often seen to influence an infant’s developmental outcomes later in life (Black *et al.*, 2016). However, infants may not be identified early enough in LMIC to effectively treat the feeding problems and to prevent future developmental delays (Samuels *et al.*, 2012). Unresolved early feeding difficulties may consequently exacerbate impaired development in at-risk populations, result in stunted growth and malnutrition, or in severe cases lead to death (Berlin *et al.*, 2009). Investigation of the relationship between feeding and developmental outcomes in infants is therefore warranted.

The aetiologies of feeding and developmental outcomes in infants are heterogeneous and complex, leading to the involvement of multiple healthcare professionals (Estrem, 2015; Borowitz and Borowitz, 2018). Each discipline focuses on a particular aspect of the feeding problem in a child-centred multidisciplinary approach (Estrem, 2015). Selective intake of food and disruptive mealtime behaviour in young children may, for example, be common problems being treated in Occupational Therapy, Speech-Language Pathology, and Psychology, but uncommon to the professions of medicine and nursing (Estrem *et al.*, 2017). Healthcare professionals may have difficulty identifying feeding difficulties in infants due to the complex relationship between biopsychosocial factors involved during feeding (Berlin *et al.*, 2009; Estrem, 2015). Upon entry into specialised care for feeding difficulties, limited literature is available regarding discipline-specific attributes of problematic feeding, and the impact on development and conversely, development and its impact on feeding (Estrem, 2015). A common framework capable of organising and analysing multiple professions’ approaches and EI goals is necessary (Guralnick, 2011).

The developmental systems approach (DSA) considers an interplay between protective and risk factors at all levels, and infants who have biological or established risks are less resilient to maintaining optimal levels of development during poor-quality family patterns of interaction (Guralnick, 2011). Due to the complexity of the factors that may be present, there is a need to conceptualise the interaction between biological and environmental factors on feeding difficulties and early childhood development in LMIC. Infants in an HIV-affected household, for example, may not receive the appropriate stimulation and care, which increases the risk of developmental delay (McDonald *et al.*, 2013). Furthermore, having an infant with a developmental delay in the household may lead to the development of stressors within the family system, which negatively influences family patterns of interaction (Guralnick, 2013). Thus, developmental difficulties may influence parent–infant interaction as well as feeding, creating a vicious cycle in infant development (Crapnell *et al.*, 2015). While this may be true, a critical source of a protective factor lies with the infants themselves (Guralnick, 2011). As a result of a genetic predisposition or the presence of nurturing care, an infants’ resilience to environmental risks may increase (Guralnick, 2011; Britto *et al.*, 2017). For example, secure infant–caregiver attachment during early childhood may protect them from the risk of developmental delay in a household where negative family patterns of interaction may be present (Guralnick, 2013). The interaction between protective and risk factors at all levels of the DSA remains complex.

The importance of early identification of developmental and feeding difficulties in infants is evident. EI programmes in primary health care settings in LMIC are overburdened due to limited numbers of healthcare professionals, as well the lack of resources and facilities to implement EI services (Samuels *et al.*, 2012). Yet, the identification of risk factors that lead to feeding difficulties and developmental delay in early childhood should be prioritised (Van der Linde *et al.*, 2015a). This would strengthen primary preventative strategies, such as developmental screening, surveillance, and intervention, in order to compensate for risks to reduce or eliminate resultant feeding or developmental delays (Van der Linde *et al.*, 2015a). Early identification of feeding problems in infants would improve the monitoring of developmental outcomes and vice versa (Barratt and Ogle, 2010). The current study aimed to determine the relationship between feeding characteristics and general developmental outcomes in infants in a South African community.

## Method

### Setting

Retrospective data were originally collected (Fuls, 2019) at a well-baby immunisation clinic in a resource-limited community in the Tshwane District, Gauteng province of South Africa in a previous study. This community is approximately 25 km^2^ with an estimated population of close to a million people (Darkey and Visagie, 2013). The majority of the population lives in informal settlements comprising mostly of self-built houses (Mashigo, 2012). The people residing in this community use the primary health care clinic as their first point of access to health care. Infants in South Africa attend an immunisation clinic at six, nine, and twelve months of age, but developmental screening is not required by law during these visits (Samuels *et al.*, 2012; National Institute for Communicable Diseases, 2016).

### Participants

The original study made use of convenience sampling to recruit infants aged six to twelve months over a period of four months. All parents/caregivers of infants aged six to twelve months attending the well-baby immunisation clinic were asked to participate in the study. There were 250 participants whose parents/caregivers provided voluntary informed consent in the previous study. In the present study, 144 complete data sets were retrospectively analysed after excluding those with missing data. Table 1 presents the participant characteristics. The majority of the sample (*n* = 81; 56.3%) were male with a mean age (standard deviation) of 8.5 months (2.2). Most of the primary caregivers were mothers (91.7%) with a Black ethnic background (100%). Many participants (72.3%) were living in an informal dwelling and most caregivers (77.1%) were unemployed.

**Table 1.** Description of participants

Description	Category	*n* = 144	%*
Gender of infant	Male	81	56.3
	Female	63	43.8
Age of infant (months)	Mean (standard deviation)	8.5 (2.2)	–
Primary caregiver	Mother	132	91.7
	Grandparents	7	4.9
	Extended family	4	2.8
	Both parents	1	0.7
Age of caregiver (years)	17–20	10	6.9
	21–30	92	63.9
	31–40	36	25.0
	41–50	6	4.2
Education of caregiver	Less than Grade 8	6	4.2
	Grade 8 to Grade 10	12	8.3
	Grade 11 to Grade 12	110	76.4
	Diploma/degree	16	11.1
Housing status	Informal dwelling	104	72.3
	Formal dwelling	40	27.7
Employment status of primary caregiver	Employed	33	22.9
	Unemployed	111	77.1
Home language	IsiZulu	21	14.6
	Sepedi	71	49.3
	Sesotho	11	7.6
	Xitsonga	12	8.3
	Other	29	20.1

* All figures are rounded off to one decimal place.

### Measures

Retrospective data were obtained using the following measures: Background information was obtained from a questionnaire comprising of 57 questions (Van der Linde *et al.*, 2015b). The questionnaire provided information about the parent/caregiver’s socioeconomic and educational status, as well as psychosocial factors, such as poor environmental stimulation and problematic parent–child interactions, possibly influencing the infant’s feeding and developmental outcomes (Berlin *et al.*, 2011). Information on the participants’ feeding history and the current development of skills were also obtained.

A screening of each participant’s feeding ability was conducted using the Montreal Children’s Hospital Feeding Scale (MCH-FS) (Ramsay *et al.*, 2011). Items in the MCH-FS included oral sensorimotor skills and appetite. Maternal concerns relating to feeding, mealtime behaviour, mealtime strategies used, and family reactions to the participant’s feeding were investigated. The MCH-FS is a valid and reliable tool that demonstrates appropriate specificity and sensitivity, and its application can be extended to the general population (Benjasuwantep *et al.*, 2015; Sanchez *et al.*, 2015; Barton *et al.*, 2017). Although the MCH-FS was developed in a high-income country with different social and cultural expectations, the maturational component of feeding development makes feeding parameters universal (Benjasuwantep *et al.*, 2015; Sanchez *et al.*, 2015). This, combined with the instinctual maternal component, further demonstrates the beneficial application of the MCH-FS as a quick screening tool in a culturally diverse population (Ramsay *et al.*, 2011; Benjasuwantep *et al.*, 2015).

An assessment of each participant’s development was conducted using the Parent’s Evaluation of Developmental Status (PEDS) tools, which includes the PEDS and Parent’s Evaluation of Developmental Status – Developmental Milestones (PEDS-DM) (Brothers *et al.*, 2008). The PEDS identifies parental/caregiver concern regarding global/cognitive, receptive language, expressive language and articulation, fine motor, gross motor, behaviour, social–emotional, and self-help skills (Glascoe, 1997). The PEDS uses an algorithm specified by pathways A–E for referral (Glascoe, 1997). Pathway A is considered a fail and E indicates a pass, whereas pathways B–D represent a referral to the PEDS-DM where a pass or fail will be determined. The PEDS-DM consists of six questions regarding the infant’s developmental milestones. Developmental domains assessed included fine motor, receptive language, expressive language, gross motor, self-help, and social–emotional skills. The PEDS and PEDS-DM demonstrate high sensitivity (75% and 82%) and specificity (80% and 83%) scores in infants from birth to 12 months (Van der Linde *et al.*, 2015b). A recent study confirmed the accuracy of the application of the PEDS and PEDS-DM as a mobile health (mHealth) tool to the South African population (Maleka *et al.*, 2016). Thus, the PEDS tools were deemed appropriate tools to use within the primary health care context of South Africa (Van der Linde *et al.*, 2015b).

### Procedure

Institutional ethical clearance was obtained (GW20180121HS). The background questionnaire and MCH-FS were administered in a structured interview with each parent/caregiver. An assessment of each participant’s general development was then conducted using the PEDS and the PEDS-DM. Data were collected from the completed measurement score sheets.

### Data analysis

The relationship between feeding and developmental outcomes was investigated by retrospectively analysing data on 144 complete data sets. Spearman’s rho was used to measure correlations between the feeding history of the participants and the PEDS and PEDS-DM outcomes. Correlations between items and raw scores from the MCH-FS, and the PEDS and PEDS-DM outcomes were also investigated. Furthermore, the phi-coefficient was used to measure associations between infants’ feeding history and development and the PEDS and PEDS-DM outcomes. A *P*-value of less than or equal to .05 was considered statistically significant.

## Results

The feeding characteristics of the sample are shown in Table 2. Interestingly, 123 (85.4%) infants had been breastfed. Bottle-feeding was initiated between zero and six months in 81 (56.3%) infants, while cup feeding was introduced between one and five months in 18 (12.5%) infants. Of the 144 infants, 103 (71.5%) had already commenced with spoon-feeding.

**Table 2.** Feeding characteristics

Description	Response	*n* = 144	%
Neonatal feeding difficulties	Yes	8	5.6
Colostrum	Yes	121	84
Type of milk given	Breastmilk Formula Mixed feeding: breastmilk and formula	76 51 17	52.8 35.4 11.8
Breastfed Duration of breastfeeding Method Length of breastfeeding session	Yes 6 months or less More than 6 months Direct breastfeeding 5 min 10 min 15 min 20–25 min	123 53 66 116 28 23 20 15	85.4 36.8 45.8 80.6 19.4 16 13.9 6.9
Bottle-feeding Duration of bottle-feeding Length of bottle-feeding session	Yes 0–6 months 7–10 months More than 10 months 5 min 10 min 15 min 20–25 min	110 81 20 10 43 13 11 5	76.4 56.3 13.9 6.9 29.9 9 7.6 3.5
Cup feeding Age of cup introduction	Yes 1–5 months 6–8 months More than 8 months	71 18 43 10	49.3 12.5 29.9 6.9
Age of solid food introduction Method Number of tablespoons per meal	4–5 months or less 6–8 months 9–10 months or more Spoon-feeding Mother’s hand 1–5 tablespoons 6–10 tablespoons	9 92 8 103 6 79 29	6.3 63.9 5.6 71.5 4.2 54.9 20.1

Seven infants (4.9%) failed the MCH-FS. Of the seven infants, five were classified as having a mild feeding difficulty, while the remaining two infants were evenly distributed between moderate and severe feeding difficulty. Item-specific results for the MCH-FS are shown in Table 3.

**Table 3.** Item-specific results of the MCH-FS

Question	Response	*n* = 144	%
How do you find mealtimes with your child?	Very difficult Easy	14 130	9.7 90.3
How worried are you about your infant’s feeding?	Not worried Very worried	130 14	90.3 9.7
How much appetite (hunger) does your child have?	Never hungry Good appetite	6 138	4.2 95.8
When does your child start refusing to eat during mealtimes?	At the beginning At the end	22 122	15.3 84.7
How long do mealtimes take for your child (in minutes)?	1–30 min 31–>60 min	139 5	96.5 3.5
How does your child behave during mealtimes?	Behaves well Acts up, makes a big fuss	133 11	92.4 7.6
Does your child gag or spit or vomit with certain types of food?	Never Most of the time	126 18	87.5 12.5
Does your child hold food in his/her mouth without swallowing?	Most of the time Never	9 135	6.2 93.8
Do you have to follow your child around or use distractions (toys, television) so that your child will eat?	Never Most of the time	120 24	83.3 16.7
Do you have to force your child to eat or drink?	Most of the time Never	13 131	9 91
How are your child’s chewing (or sucking) abilities?	Good Very poor	134 10	93.1 6.9
How do you find your child’s growth?	Growing poorly Growing well	3 141	2.1 97.9
How does your child’s feeding influence your relationship with him/her?	Very negatively Not at all	4 140	2.8 97.2
How does your child’s feeding influence your family relationship?	Not at all Very negatively	142 2	98.6 1.4

Results from the PEDS indicated that 47 (28.5%) infants failed (path A–D) the developmental assessment. It was further shown that 58 (40.3%) infants failed the PEDS-DM. The final outcome of the PEDS tools indicated that 58 (40.3%) infants failed the developmental screening. The distribution of the participants according to responses given in the PEDS and PEDS-DM is shown in Tables 4 and 5.

**Table 4.** Developmental domain-specific caregiver concerns according to PEDS

PEDS area	Indicating ‘Yes’ to concerns for PEDS area	
	*n* = 144	%
Expressive language and articulation	6	4.2
Receptive language	5	3.5
Fine motor	2	1.4
Gross motor	0	0
Behaviour	1	0.7
Social–emotional	3	2.1
Self-help	1	0.7
School	5	3.5

**Table 5.** Developmental domain-specific ‘fail’ outcomes according to PEDS-DM

PEDS-DM area	*n* = 144	%
Expressive language	11	7.6
Receptive language	1	0.7
Fine motor	21	14.6
Gross motor	10	7
Social–emotional	14	9.7
Self-help	12	8.3

Infants who were breastfed (*ø* = 0.013; *P* = 0.010) and received both colostrum (*ø* = 0.022; *P* = 0.020) and breastmilk (*ø* = 0.009; *P* = 0.012) showed a significant association with developmentally appropriate receptive language and self-help outcomes on the PEDS and PEDS-DM, respectively. A decrease in the age of solid food introduction led to a higher possibility of failed fine motor outcome (*P* = 0.015) on the PEDS-DM. The younger the infant at age of cup drinking introduction, the stronger the association with possible expressive language and articulation concerns (*P* = 0.030) on the PEDS. A longer breastfeeding duration was strongly correlated with failed self-help outcomes (*P* = 0.009). Conversely, a longer breastfeeding duration was associated with developmentally appropriate expressive language skills (*P* = 0.026) on the PEDS-DM. The results further indicated that the longer the participants were bottle-fed, the stronger the association of parental concern for school performance in the future (*P* = 0.039) and failed self-help outcomes (*P* = 0.004) on the PEDS-DM. In addition to this, longer durations of bottle-feeding (*P* = 0.043) and an increase in the number of spoons used per meal (*P* = 0.022) have a stronger association with the increased likelihood of participants being closer to path A on the PEDS (i.e., a fail and subsequent referral to PEDS-DM).

The results indicated that possible behavioural (*P* = 0.007), social–emotional (*P* = 0.001), expressive language and articulation (*P* = 0.033), and future school performance (*P* = 0.001) concerns on the PEDS, as well as failed self-help outcomes (*P* = 0.036) on the PEDS-DM, were significantly associated with higher MCH-FS raw scores. Higher MCH-FS scores are indicative of an increased likelihood of presenting with feeding difficulties according to the MCH-FS. Furthermore, a negative correlation between the MCH-FS raw score and the PEDS pathway (*P* = 0.001) revealed that the higher the MCH-FS raw score was, the more likely the participant was closer to path A on the PEDS. Item-specific results from the MCH-FS revealed that food refusal at the beginning of meals was associated with fine motor (*P* = 0.013) and social–emotional (*P* = 0.005) concerns on the PEDS. Acting up and making a big fuss during mealtimes correlated with gross motor (*P* = 0.001) and behavioural (*P* = 0.034) concerns, while the use of distractions (i.e., toys or television) during mealtimes correlated with social–emotional concerns (*P* = 0.011) on the PEDS. The presence of gagging, spitting, or vomiting showed an association with behavioural (*P* = 0.001) and social–emotional concerns (*P* = 0.001), as well as concerns for future school performance (*P* = 0.001) on the PEDS, and failed expressive language (*P* = 0.026) and gross motor (*P* = 0.038) outcomes on the PEDS-DM. A relationship between pocketing or holding food in the mouth, and behavioural (*P* = 0.013) and social–emotional (*P* = 0.023) concerns on the PEDS and failed fine motor outcomes (*P* = 0.038) on the PEDS-DM, was also revealed. Force feeding and poor sucking and chewing abilities were associated with behavioural (*P* = 0.001; *P* = 0.022) and social–emotional (*P* = 0.001; *P* = 0.001) concerns, as well as concern for future school performance (*P* = 0.019; *P* = 0.001), respectively.

## Discussion

Although only 4.9% of infants were referred on the feeding screening, it was evident that caregiver concern for developmental outcomes was significantly higher (40.3%). This is noteworthy as both are based on caregiver report (Ramsay *et al.*, 2011; Van der Linde *et al.*, 2015b). The discrepancy between caregiver report of feeding and developmental outcomes may be due to the non-specificity and heterogeneity of red flags in early feeding development leading to misinterpretation by caregivers (Estrem *et al.*, 2017).

It was found that developmentally appropriate receptive language and self-help outcomes were significantly associated with infants who were breastfed (*ø* = 0.013; *P* = 0.010) and given both colostrum (*ø* = 0.022; *P* = 0.020) and breastmilk (*ø* = 0.009; *P* = 0.012). Our findings further suggested that breastfeeding for more than six months may be associated with developmentally appropriate expressive language skills (*P* = 0.026). Evidence supports this finding as breastfeeding for more than 12 months may be associated with developmentally appropriate language and cognitive skills (Iqbal *et al.*, 2018). These findings reveal that breastfeeding may act as a protective factor for language and adaptability in infants in LMIC. This is valuable as breastfeeding forms part of nurturing care provided by a mother, which supports the development of key brain areas and promotes developmental adaptation (Britto *et al.*, 2017). Thus, breastfeeding increases infant’s internal resilience to environmental risks and therefore reduces the risk of later developmental delays (Guralnick, 2013; Britto *et al.*, 2017). Conversely, poor self-help outcomes (*P* = 0.009) were associated with infants breastfeeding for a longer period. Research indicates that attachment security plays a role in the infants’ ability to request help or independently interact with their environment (Rispoli *et al.*, 2013). However, it has also been established that breastfeeding does not reduce infants’ temperamental dependency or level of clinginess, which may account for poor self-help outcomes experienced by infants who were breastfed for more than six months (Gibbs *et al.*, 2018).

Results revealed that early introduction of solid foods may be associated with poor fine motor outcomes (*P* = 0.015). Early introduction of cup feeding was also found to be a predictor of possible expressive language and articulation difficulties (*P* = 0.030). These associations may be related to the unique progression of oral function during transitional feeding (Borowitz and Borowitz, 2018). There is a close relationship between motor development and oral-feeding ability (Delaney and Arvedson, 2008). Sufficient fine motor control, occurring between five and six months of age, is required for picking up food by hand or with a spoon in order to meet transitional feeding milestones (Borowitz and Borowitz, 2018). Furthermore, there is preliminary evidence supporting the simultaneous development of speech and feeding skills where a deficit in one area, results in deficits in the other (Dent, 2018). In the current study, 29.9% of the participants commenced with cup feeding between six and eight months; but, it is not until 11 months of age where an infant is able to drink from a cup independently and efficiently (Borowitz and Borowitz, 2018). The immaturity of the oral-feeding mechanism during early cup introduction may explain the associated expressive language and articulation concerns in this population.

The findings suggest that a longer duration of bottle-feeding is linked to poor self-help outcomes (*P* = 0.004) and concern for future school performance (*P* = 0.039). In addition to this, longer durations of bottle-feeding (*P* = 0.043) as well as an increase in the number of spoons used per meal (*P* = 0.022) may be associated with poor developmental outcomes. It is established that poor mother–infant attachment is correlated with suboptimal developmental outcomes (Branjerdporn *et al.*, 2017). It may be possible that due to the prolonged duration of bottle-feeding, these infants were not able to bond optimally with their caregiver and benefit from the developmental gains associated with this (Iqbal *et al.*, 2018). To our knowledge, there is no evidence linking the duration of bottle- or spoon-feeding to poor developmental outcomes. These preliminary findings may offer insight into the importance of feeding practices in early and late infancy and their influence on development (Iannotti *et al.*, 2016).

There were significant correlations between acting up (*P* = 0.034), gagging, spitting, or vomiting (*P* = 0.001), pocketing (*P* = 0.013), the use of force feeding (*P* = 0.001), and poor sucking and chewing abilities (*P* = 0.022) with behavioural difficulties in this sample. The relationships between feeding and behavioural characteristics may be typical of this population transitioning from a liquid to solid diet (Kerzner *et al.*, 2015). Mealtimes become structured around allowing the infant to explore safely and finding a balance between autonomy and dependency (Delaney and Arvedson, 2008). Poor behaviour may prompt inappropriate feeding strategies, aggravating behavioural issues, and causing a long-term problem (Kerzner *et al.*, 2015).

An explanation for the strong relationship between social–emotional difficulties and food refusal (*P* = 0.005), the use of distractions (*P* = 0.011), poor sucking and chewing (*P* = 0.001), gagging, spitting, or vomiting (*P* = 0.001), pocketing (*P* = 0.023), and force feeding (*P* = 0.001) during mealtimes may be related to the misinterpretation of typical feeding development (Kerzner *et al.*, 2015). As infants exert more control over their environment, caregivers may misinterpret these feeding characteristics as caused by possible social–emotional difficulties, whereas these behaviours may be typical of the six- to twelve-month population (Delaney and Arvedson, 2008).

A relationship between fine motor difficulties and food refusal at the beginning of meals (*P* = 0.013) and pocketing (*P* = 0.038), and gross motor difficulties with acting up (*P* = 0.001) and gagging, spitting, or vomiting (*P* = 0.038) was found. The following explanations for these relationships are only speculative. Sensory over-reactions (such as gagging, spitting, or vomiting) occur as sensory tolerances emerge and align with the development of oral motor skills (Van den Engel-Hoek *et al.*, 2014). The presence of such a maladaptive mealtime cycle may indicate transactional development between the caregiver and the infant, negatively impacting the infant’s development (Guralnick, 2013; Estrem *et al.*, 2017). Conversely, poor infant development such as delayed fine or gross motor skills may create a maladaptive mealtime cycle, continuing this negative pattern (Crapnell *et al.*, 2015). Another possible explanation may be that these feeding characteristics are suggestive of a relationship between transitional feeding and neuro-motor maturity (Delaney and Arvedson, 2008). Further research in the typical population is needed in this regard.

A significant association between food transition difficulties and failed expressive language outcomes (*P* = 0.026) and an increased concern for future school performance (*P* = 0.001; *P* = 0.019; *P* = 0.001) were shown. A previous study found that children with a language impairment had a history of food selectivity and food transition difficulties (Malas *et al.*, 2017). This association may be due to food transition difficulties negatively influencing parent–infant interactions with a cascading effect on language development and cognitive competence (Guralnick, 2013; Malas *et al.*, 2017).

### Implications for practice and policy

The current study advocates for EI healthcare professionals to be trained to use the DSA (Guralnick, 2005) to increase the awareness of the role of environmental factors on infants’ developmental progression. Furthermore, the findings indicated that the awareness of typical feeding development should be raised among caregivers so that this may be contrasted with developmental concerns. This would ensure that caregivers seek EI services timeously. The findings further provided evidence for a national change in public policy on feeding and developmental screening, surveillance, and monitoring in South Africa and LMIC. The provision of feeding and developmental screening, surveillance, and monitoring services would have subsequent influences on the implementation of secondary and tertiary preventive strategies. EI healthcare professionals should therefore participate in advocacy activities for a change in public policy for EI services in LMIC.

### Strengths and limitations

There were several strengths and limitations of the present study. Although a retrospective design allowed for the analysis of a large sample previously collected, it also presented with the challenge of missing data (Neuman, 2014). The retrospective design also limited the present study’s ability to investigate correlations between clinical assessment and caregiver report as a small number of infants (*n* = 7) were referred for an in-depth clinical assessment. However, the use of caregiver report allowed for a preliminary investigation of caregiver knowledge in a resource-limited community in South Africa.

### Recommendations for future research

The findings indicated that breastfeeding may be a protective factor for language development in LMIC. The interrelationship between feeding and communication development is complex and requires continuous research (Delaney and Arvedson, 2008). Future studies should therefore investigate the influence of breastfeeding on other language domains in early and late infancy. It is further recommended that future studies investigate caregiver knowledge and perceptions of transitional feeding in order to examine how this may influence infant development. An investigation into prevention strategies and the role of the EI team in primary health care should also be explored in order to identify the roles, perceptions, and knowledge of EI healthcare professionals. The use of a prospective research design with a larger sample is additionally recommended to strengthen the current study’s findings. Furthermore, future studies should consider the use of clinically validated diagnostic feeding and developmental assessment tools to comprehensively investigate the relationship between feeding and developmental outcomes in late infancy.

## Conclusion

This study found significant associations between certain feeding characteristics and the developmental outcomes in infants aged six to twelve months in a resource-limited South African community. The findings suggest that breastfeeding may be a protective factor for language development and adaptability in this population. Further research is necessary. In LMIC where healthcare services are overburdened, caregivers become the agents of change for their infants (Samuels *et al.*, 2012). The results of this study may be used to advocate for the education of caregivers and for the provision of EI services in resource-limited settings. Caregivers may benefit from education on the identification of behavioural red flags regarding their infant’s feeding so that they may contrast these with typical development. Clinicians in primary health care may use these findings to provide parent guidance and developmental surveillance regarding normal feeding development during infancy so that caregivers are able to differentiate between typical development and developmental concerns. This would strengthen primary preventative strategies in order to compensate for prevalent risk factors present in LMIC and improve monitoring of feeding and developmental outcomes to relieve the strain on overburdened EI services.
